# Percutaneous transcatheter arterial embolization in haemodynamically stable patients with blunt splenic injury

**DOI:** 10.2478/v10019-010-0011-2

**Published:** 2010-03-18

**Authors:** Peter Popovic, Dragoje Stanisavljevic, Miran Jeromel

**Affiliations:** 1 Institute of Radiology, University Medical Centre Ljubljana, Ljubljana, Slovenia; 2 Clinical Department for Surgery, University Medical Centre Ljubljana, Ljubljana, Slovenia

**Keywords:** splenic trauma, treatment, angiography, percutaneous transcatheter embolization

## Abstract

**Background:**

The nonoperative management of the blunt splenic injury in haemodynamically stable patients has become an accepted treatment in recent years. We present a case of the blunt splenic injury successfully treated by supraselective embolization with microspheres.

**Case report.:**

A young hockey player was brought to the Emergency Department with the history of blunt abdominal trauma 2 h earlier. A Grade III splenic injury with haemoperitoneum was diagnosed on sonographic evaluation and the patient was treated with the selective distal splenic artery embolization with microspheres. Postprocedural ultrasound and computed tomography follow-up a year later revealed only a small area of parenchymal irregularity.

**Conclusions:**

The percutaneous splenic arterial embolization has a major role in the management of traumatic splenic injuries. Embolization is particularly beneficial in injuries of grade III or higher.

## Introduction

Spleen injuries are most commonly associated with blunt abdominal trauma and represent a potentially life-threatening condition. The management of splenic trauma is still controversial, but there have been major changes over the last three decades. In the past, any damaged spleen was surgically removed to avoid a delayed rupture. The increased susceptibility of the patient to infection after splenectomy – in particular, the risk of overwhelming, potentially fatal postsplenectomy sepsis – motivated physicians to favour splenic preservation procedures.^1^ Nonsurgical management (NOM) with bed rest and observation has traditionally been the treatment of choice for the splenic injury in paediatric patients. Although the nonsurgical management of stable blunt splenic injuries in adults has gained popularity in recent years, the initial choice of surgical versus nonsurgical management remains controversial. However, embolization is also wildly used for another indications with much less controversial results.^2^,^3^ The controversy of the splenic arterial embolization has been attributed to the relatively high failure rate of such a treatment (10–31%), with a resultant need for secondary splenectomy, and to the potential of missing other intra-abdominal injures that require laparotomy. The splenic transcatheter arterial embolization (TAE) has been proposed to reduce the risk of nonsurgical management failure in adults and children. Sclafani *et al*. reported a series of cases in which NOM by means of the transcatheter arterial embolization was successful in 91% of hemodynamically stable patients, and the splenic function was preserved in all patients who underwent TAE.^1^ The most widely accepted indication for TAE is evidence of the arterial injury on a computed tomography (CT) scan. In cases of the arterial injury, embolization is performed with microcoils or gelfoam particles as distally as possible, in a small arterial branch that supplies the segment in which the extravasation is detected, to preserve perfusion to the remaining splenic parenchyma.^1^,^2^ We report on a patient with blunt splenic trauma who was successfully treated by the supraselective embolization with microspheres.

## Case report

**FIGURE 1:**
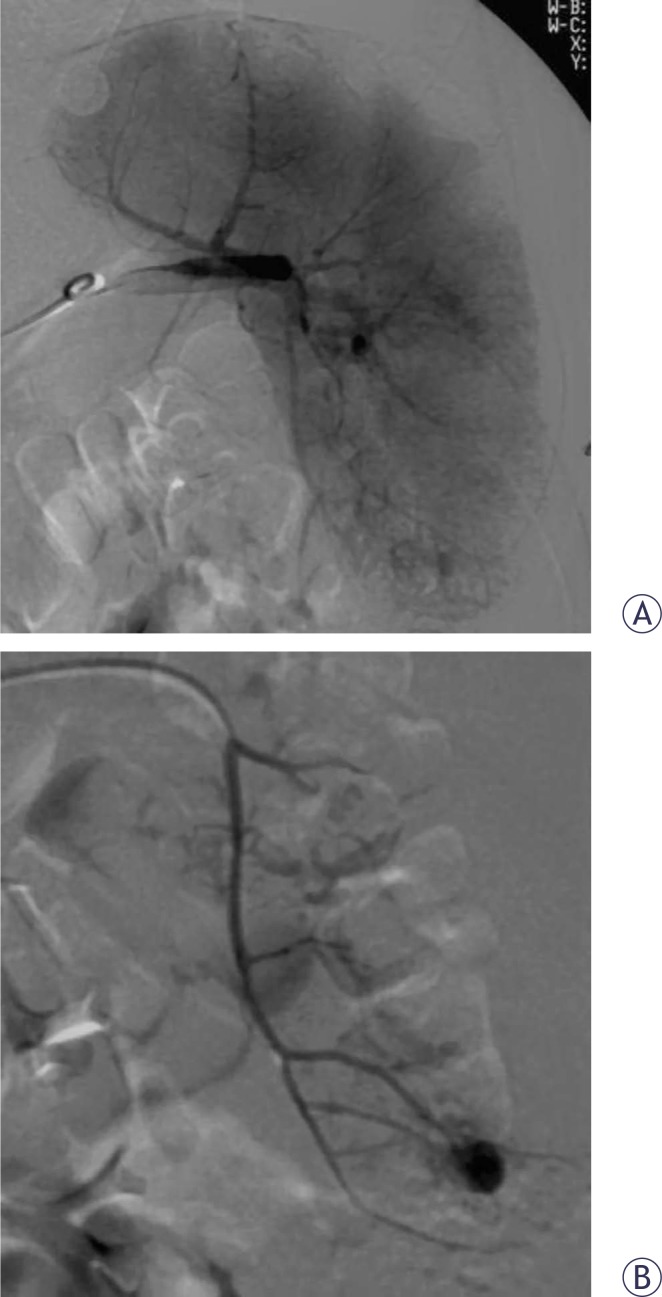
A. Selective splenic artery embolization in a 20-year-old man with blunt abdominal trauma. Splenic angiography obtained before the embolization procedure shows active extravasation from distal branch of splenic artery. B. Supraselective splenic angiography obtained with microcatheter – the extravasation is better seen.

**FIGURE 2:**
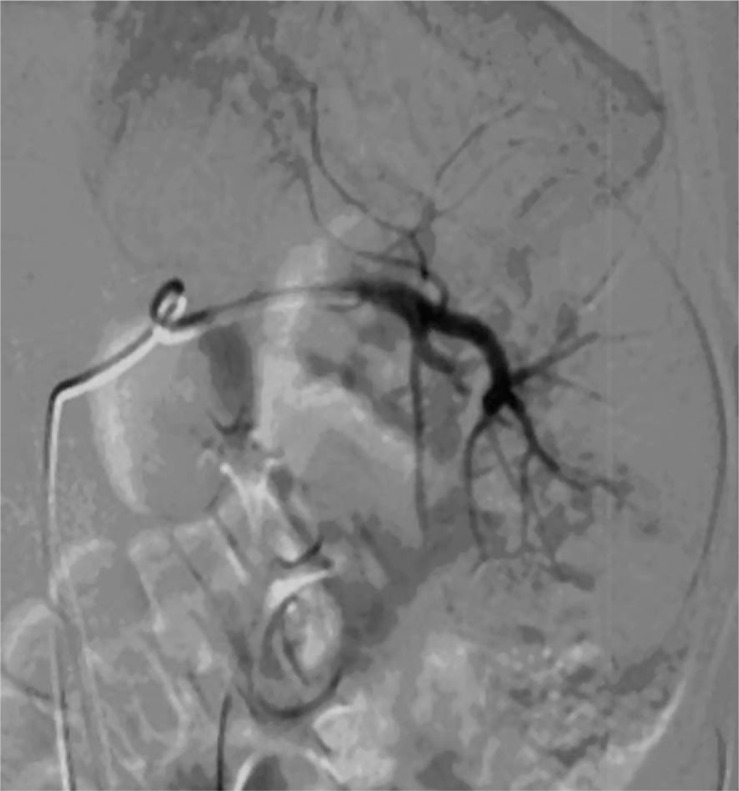
Splenic angiography obtained after the selective embolization with Bead Block microspheres (diameter range, 700–900 μm) via a Progreat 2.8 Fr microcatheter shows a complete haemostasis. No extravasation is seen.

**FIGURE 3:**
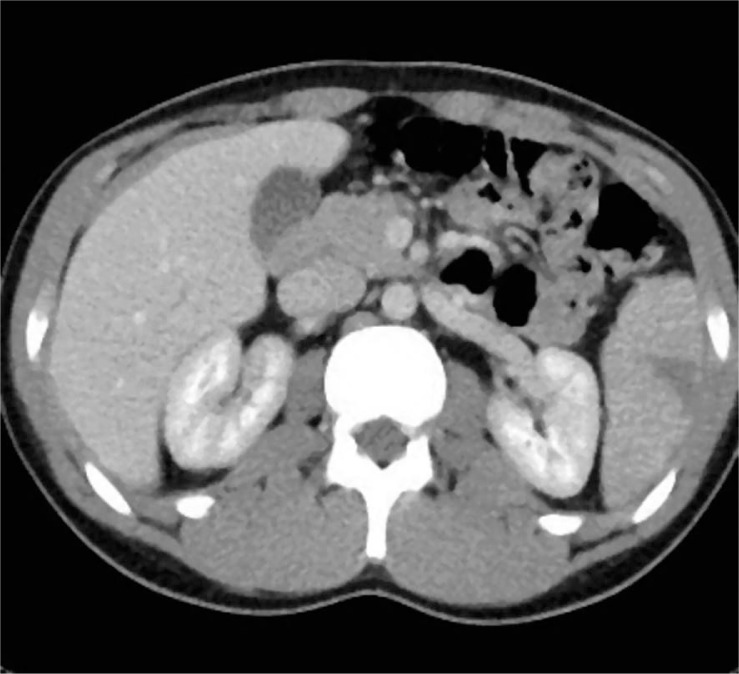
Transverse CT scan, obtained one month after the embolization shows small area of parenchymal infarct.

A 20-year-old hockey player presented with blunt trauma to the left upper abdomen. The abdominal ultrasound revealed a small amount of free fluid around the spleen (haemoperitoneum). A small intraparenchimal splenic haematoma and a laceration 4 cm in depth were seen in the lower pole of the spleen. The repeated abdominal ultra-sound (performed 4 h later) revealed an increased amount of free abdominal fluid (around 1000 cm^3^). The splenic haematoma had also increased in size (measuring 5.5 cm in diameter). The patient was haemodynamically stable. His blood pressure was 110/85 mmHg with a heart rate of 90 beats/min. A multidisciplinary decision for the nonoperative treatment – percutaneous embolization – was reached. An urgent angiogram was performed to identify and possibly also treat the source of bleeding. The informed, written consent of the patient was obtained before the procedure for both diagnostic angiography and possible embolization. The selective catheterisation of the splenic artery with a 5F Sidewinder catheter (Cordis, Miami, FL, USA) demonstrated the extravasation of contrast media from the distal branch of the splenic artery (Figure 1A,B). A decision to perform the distal splenic artery embolization was made. Supraselective catheterisation and embolization with 500–700 μm Bead Block (Biocompatibles, Farnham, Surrey, UK) microspheres via a Progreat 2.8 Fr microcatheter (Terumo, Leuven, Belgium) were performed. A postprocedural splenic arteriogram showed the successful embolization (Figure 2). Follow-up ultrasound and CT examination revealed a small area of infarction at the site of the embolization (Figure 3). The patient was stable and discharged from hospital two weeks later. A postprocedural follow-up a year after the procedure revealed only a small area of parenchymal irregularity.

## Discussion

The nonoperative management of blunt splenic injuries is the treatment modality of choice in haemodynamically stable adults and paediatric patients regardless of the severity of the injury. The embolization is a useful adjunct in the nonoperative management of patients who continue to bleed (Eastern Association for the Surgery of Trauma, Trauma Practice Management Guidelines, 2003). These guidelines are now accepted in most modern trauma centres. Contrast-enhanced CT has been shown to be highly accurate in diagnosing acute splenic injuries. It enables the classification of the splenic injury according to severity, for which the Organ Injury Scale for the spleen (American Association for the Surgery of Trauma-AAST) is a widely accepted grading system.^4^–^6^ The scale is as follows: Grade I – subcapsular haematoma of less than 10% of surface area or capsular tear of less than 1 cm in depth; Grade II – subcapsular hematoma of 10–50% of surface area or intraparenchymal haematoma of less than 5 cm in diameter or laceration of 1–3 cm in depth and not involving trabecular vessels; Grade III – subcapsular haematoma of greater than 50% of surface area (or expanding and ruptured subcapsular or parenchymal haematoma) or intraparenchymal hematoma of greater than 5 cm (or expanding) or laceration of greater than 3 cm in depth (or involving trabecular vessels); Grade IV – laceration involving segmental or hilar vessels with the devascularisation of more than 25% of the spleen; Grade V – shattered spleen or hilar vascular injury. Patients with AAST grade I or II splenic injuries and no associated splenic vascular injuries can be managed with just a simple observation. Those who are found to have one of the previously mentioned CT findings indicative of angioembolization — including AAST grade III–V splenic injury, active contrast extravasation or vascular injury of the spleen (pseudoaneurysm or A–V fistula) — should proceed to angiography and splenic embolization.^4^,^6^

The objective of the splenic arterial embolization is to improve the results of the nonoperative management.^7^ The embolization is performed via percutaneous access (usually via the common femoral artery). There are two methods regarding the splenic artery embolization. The decision about which method to use depends on angiographic findings. The distal splenic artery embolization is the method of choice for the management of a haemorrhage which originates from a distal branch of the splenic artery. This type of embolization is usually performed with microcoils and/or gelatin sponge pledgets that are injected through a microcatheter.^2^,^8^–^11^ This technique achieves haemostasis to the injured parts while preserving the perfusion to the remainder of the spleen.

When a haemorrhage persists in spite of the distal embolization or the patient is at high risk of secondary spleen rupture (injury Grade III or higher), a more proximal splenic artery embolization is performed which reduces the pressure in the splenic parenchyma.^5^,^9^–^13^ This type of embolization is usually performed with microcoils inserted in the middle segment of the splenic artery. Coils inserted at this site allow the reconstitution of blood supply through collateral vessels (short gastric and gastroepiploic, transgastric and transpancreatic arteries). The proximal embolization has been shown to be associated with less frequent and smaller infarcts than the distal embolization. It does not affect spleen anatomy or immune function.^14^ The success of the splenic arterial embolization is defined by the splenic salvage rate.

The Quality Improvement Guidelines of the Society of Interventional Radiology reports a success rate between 87–100%.^4^,^11^,^13^

Major postprocedural complications are splenic abscess and infarct. Postembolization CT shows splenic infarcts in two thirds of patients after the proximal embolization and in all cases after the distal embolization.^14^ The reported rate of the splenic abscess after the proximal or distal embolization is 3%.^10^,^13^ Other relatively rare complications include coil migration, iatrogenic vascular injury and missed injuries to the diaphragm or pancreas.

Grading of the spleen injury in our case was not based on CT but on the sonographic evaluation. It was estimated as a Grade III injury (intraparenchymal haematoma greater than 5 cm and laceration greater than 3 cm in depth). The patient was haemodinamically stable but continued to bleed, and, therefore, the decision to perform embolization was made. Arteriography revealed bleeding from a small distal branch of the splenic artery. The successful distal splenic artery embolization with microspheres was performed. An early postprocedural follow up with CT and ultrasound was performed, revealing only a small area of parenchymal infarct (less than 2 cm in diameter). A postprocedural follow-up a year after the procedure revealed only a small area of parenchymal irregularity.

## Conclusions

The percutaneous splenic arterial embolization has a major role in the management of traumatic splenic injuries. The embolization is particularly beneficial in injuries of AAST grade III or higher. Microspheres can be used as an alternative to microcoil or gelfoam particles for the distal splenic artery embolization.
